# Large Comparative Analyses of Primate Body Site Microbiomes Indicate that the Oral Microbiome Is Unique among All Body Sites and Conserved among Nonhuman Primates

**DOI:** 10.1128/spectrum.01643-21

**Published:** 2022-05-19

**Authors:** Abigail E. Asangba, Lawrence Mugisha, Joshua Rukundo, Rebecca J. Lewis, Ali Halajian, Liliana Cortés-Ortiz, Randall E. Junge, Mitchell T. Irwin, Johan Karlson, Andrew Perkin, Mrinalini Watsa, Gideon Erkenswick, Karen L. Bales, Dorothy L. Patton, Anna J. Jasinska, Eduardo Fernandez-Duque, Steven R. Leigh, Rebecca M. Stumpf

**Affiliations:** a Department of Anthropology, University of Illinois at Urbana-Champaign, Urbana, Illinois, USA; b Carl R. Woese Institute for Genomic Biology, University of Illinois at Urbana-Champaign, Urbana, Illinois, USA; c Ecohealth Research Group, Conservation & Ecosystem Health Alliance (CEHA), Kampala, Uganda; d Department of Wildlife & Aquatic Animal Resources, College of Veterinary Medicine, Animal Resources & Biosecurity (COVAB), Makerere University, Kampala, Uganda; e Chimpanzee Sanctuary and Wildlife Conservation (Chimpanzee Trust), Ngamba Island, Uganda; f Department of Anthropology, University of Texas at Austin, Austin, Texas, USA; g Research Administration and Development, University of Limpopo, Sovenga, South Africa; h Department of Ecology and Evolutionary Biology, University of Michigan, Ann Arbor, Michigan, USA; i Columbus Zoo and Aquarium, Powell, Ohio, USA; j Department of Anthropology, Northern Illinois University, DeKalb, Illinois, USA; k Tanzania Forest Conservation Group and Nocturnal Primate Research Group, Dar es Salaam, Tanzania; l San Diego Zoo Wildlife Alliance, San Diego, California, USA; m Field Projects International, Escondido, California, USA; n Division of Infectious Diseases, Department of Medicine, Washington University School of Medicine, St. Louis, Missouri, USA; o Department of Psychology, University of California Davis, Davis, California, USA; p Department of Obstetrics and Gynecology, University of Washington, Seattle, Washington, USA; q Division of Infectious Diseases, Department of Medicine, School of Medicine, University of Pittsburgh, Pittsburgh, Pennsylvania, USA; r Department of Molecular Genetics, Institute of Bioorganic Chemistry, Polish Academy of Sciences, Poznan, Poland; s Department of Anthropology, Yale University, New Haven, Connecticut, USA; t Department of Anthropology, University of Colorado—Boulder, Boulder, Colorado, USA; u Kanyanchu River Chimpanzee Project and Research Collaborative, Bigodi, Uganda; v Program in Ecology, Evolution and Conservation Biology, University of Illinois at Urbana-Champaign, Urbana, Illinois, USA; w Notre Dame Institute for Advanced Study, University of Notre Dame, Notre Dame, Indiana, USA; University of Thessaly

**Keywords:** microbiome, nonhuman primates, variation

## Abstract

The study of the mammalian microbiome serves as a critical tool for understanding host-microbial diversity and coevolution and the impact of bacterial communities on host health. While studies of specific microbial systems (e.g., in the human gut) have rapidly increased, large knowledge gaps remain, hindering our understanding of the determinants and levels of variation in microbiomes across multiple body sites and host species. Here, we compare microbiome community compositions from eight distinct body sites among 17 phylogenetically diverse species of nonhuman primates (NHPs), representing the largest comparative study of microbial diversity across primate host species and body sites. Analysis of 898 samples predominantly acquired in the wild demonstrated that oral microbiomes were unique in their clustering, with distinctive divergence from all other body site microbiomes. In contrast, all other body site microbiomes clustered principally by host species and differentiated by body site within host species. These results highlight two key findings: (i) the oral microbiome is unique compared to all other body site microbiomes and conserved among diverse nonhuman primates, despite their considerable dietary and phylogenetic differences, and (ii) assessments of the determinants of host-microbial diversity are relative to the level of the comparison (i.e., intra-/inter-body site, -host species, and -individual), emphasizing the need for broader comparative microbial analyses across diverse hosts to further elucidate host-microbial dynamics, evolutionary and biological patterns of variation, and implications for human-microbial coevolution.

**IMPORTANCE** The microbiome is critical to host health and disease, but much remains unknown about the determinants, levels, and evolution of host-microbial diversity. The relationship between hosts and their associated microbes is complex. Most studies to date have focused on the gut microbiome; however, large gaps remain in our understanding of host-microbial diversity, coevolution, and levels of variation in microbiomes across multiple body sites and host species. To better understand the patterns of variation and evolutionary context of host-microbial communities, we conducted one of the largest comparative studies to date, which indicated that the oral microbiome was distinct from the microbiomes of all other body sites and convergent across host species, suggesting conserved niche specialization within the Primates order. We also show the importance of host species differences in shaping the microbiome within specific body sites. This large, comparative study contributes valuable information on key patterns of variation among hosts and body sites, with implications for understanding host-microbial dynamics and human-microbial coevolution.

## INTRODUCTION

The primate body is home to diverse microbial communities, collectively known as the microbiome. While microbial communities inhabit multiple body sites, most of our understanding of microbes and their interactions with the host is based on work focused on the gastrointestinal tract (gut). In nonhuman primates (NHPs), factors like host phylogeny (1–4), host physiology (5), diet (6, 7), habitat quality (8, 9), and social interactions (10–12) have been shown to be associated with the composition and structure of the gut microbiome. The impacts of these factors result in substantial variations in microbial communities both within and between individuals and species, with significant impacts on the hosts (1–14). For instance, wild black howler monkeys reportedly obtain increased energy production from microbes during periods of consumption of low-energy foods (7). In humans, microbial variations are also linked to several metabolic, autoimmune, and infectious diseases. Examples include the association between low gut microbial diversity and obesity (15) and inflammatory bowel disease (16), high vaginal diversity and bacterial vaginosis (17), and disruption in native microbiota and susceptibility to pathogens like Clostridium difficile (18).

To date, most host-microbiome studies focus on a single body site within a single host species (7, 8, 19). Some studies incorporate multiple host species (4, 5, 9, 14, 20, 21), and a few extend their scope to multiple body sites within a single host species (22–26). Rare are investigations of multiple body sites among multiple host species (but see reference 27). Intra- and interhost species microbiome comparisons have helped to elucidate factors impacting microbial variation, niche differentiation, and microbial function but have also led to contrasting and confounding conclusions regarding factors (e.g., host genetics, environment, and diet) influencing the composition of the host microbiome within and between hosts and to what extent they exert their influence. For example, some studies conclude that diet is the major determinant impacting gut microbial community composition (7, 15, 19), while other studies support host-specific factors (1–5) or the environment (19) as the predominant factor. While a study’s conclusions regarding the determinants of microbial composition may hold within a particular body site or host, contrasting findings among studies can obfuscate and hinder our understanding of host-microbiome dynamics and patterns of variation. For example, the main determinants (e.g., diet) found in one study may apply on a granular level to that site and system and lead to conclusions regarding the primary driver of microbial variation, but such conclusions are not applicable to other hosts, body sites, or larger-scale comparisons, thus confounding our ability to draw clear conclusions and to apply this knowledge to enhance our evolutionary, ecological, and/or health perspectives. By focusing on larger-scale comparative host-microbial dynamics, we may discern more of this complicated picture.

Important gaps in our knowledge include what factors influence microbial community compositions within and among multiple body sites and host species and to what extent. For example, do host microbiomes group more by host species (suggesting host-specific/genetic factors) or by the composition and/or function of the body site? Do body site microbiomes share similar patterns of diversity and richness across host species despite extensive variation in host genetics, sociality, diet, and environment?

To characterize hierarchical levels of microbial variation and their potential determinants and assess how niche specialization and host species differences are related to microbial communities, this study compares 898 samples from eight distinct body sites (stool, rectum, mouth, ear, nose, vagina, penis, and axilla) among 17 phylogenetically diverse wild (*n* = 12), semicaptive (*n* = 1), and captive (*n* = 4) nonhuman primate species, representing, to our knowledge, the largest comparative study of microbial diversity across primate host species and body sites. Our objective was to describe patterns of microbiome variation among multiple body sites and host species within the order Primates. Specifically, we aimed to assess (i) microbiome variations within and among different body sites across multiple host species and (ii) microbiome variations within and among different body sites of individuals within the same host species. If niche specialization is substantially related to the observed differences, then (i) microbial communities would be expected to cluster more by body site than by host species and (ii) microbial communities sampled at the same body site within the same host species would be expected to cluster more closely together than microbial communities sampled from different body sites of the same individual. Alternatively, host-specific differences may better explain observed differences in microbial communities than body site, such that microbial communities cluster predominantly by host and by host species. Importantly, patterns of variation among microbial communities may be expressed differently depending on the body site and/or host species, such that these cross-site, cross-host species comparisons may yield a mosaic of potential determinants of variation. Such large, diverse comparisons offer the potential for novel insight into patterns and levels of microbial variation and the factors influencing microbial communities to increase our understanding of the magnitude of these effects across diverse body sites and host species and provide new insights into host-microbial dynamics and patterns of host-microbial coevolution.

## RESULTS

### Sequencing result summary.

To characterize the microbiome-based distinctions among multiple body sites and hosts, we analyzed 925 samples from 17 nonhuman primate species (Table 1; Table S1 in the supplemental material). After filtering, denoising, and chimera removal from all reads, we obtained 15,933,763 reads (mean number of reads per sample ± standard deviation [SD], 17,226 ± 7,986) (Table S2, All Samples). We also present further breakdowns of the sequencing results by host species (Table S2, Host Species) and sample type (Table S2, Sample Type). The results of our rarefaction analysis informed the selection of a sampling depth of 1,500 reads per sample, leading to the filtering of samples with fewer reads (Fig. S1), with 898 samples remaining.

**TABLE 1:** Summary of samples used in this study

Primate	Species	Location	Yr collected	Sample types^*a*^	No. of samples	Clade	Habitat
Chimpanzee	Pan troglodytes schweinfurthii	Ngamba Island, Uganda	2011	E, O, P, R, V	142	Hominoid	Semicaptive
Gibbon	Hoolock leuconedys	Gibbon Conservation Center, CA, USA	2016	E, N, O, P, R, a	10		Captive
Macaque	Macaca nemestrina	Washington National Primate Research Center, WA, USA	2011	F, R, V	112	Cercopithecoid	Captive
Sooty mangabey	Cercocebus atys	Yerkes National Primate Research Center at Emory University, GA, USA	2007–2008	A, O, R, V	96		Captive
Vervet Monkey	Chlorocebus aethiops *sabaeus*	Wake Forest University Primate Center, NC, USA	2009	R, V	74		Captive
		St. Kitts Island	2010	R, V	37		Wild
	Chlorocebus pygerythrus	Mogala, South Africa	2015 to 2016	O, N, P, R, V	40		Wild
Samango monkey	Cercopithecus albogularis	Louis Trichardt, South Africa	2016	E, N, O, R, V	7		Wild
Galago	Otolemur crassicaudatus	Mokopare, South Africa	2016	E, N, O, P, R	5	Strepsirrhini	Wild
	Galago moholi			E, O, R	3		Wild
	Otolemur garnettii	Kiwengwa Zanzibar, FR, Tanzania	2011	O, P, R, V	22		Wild
Sifaka	Propithecus verreauxi	Kirindy Mitea National Park, Madagascar	2010 to 2012	O, P, R, V	79		Wild
	Propithecus diadema	Tsinjoarivo-Ambalaomby Protected Area		E, O, R	34		Wild
Owl monkey	Aotus azarae	Guaycolec, Formosa Province, Argentina	2011	R, V	9	Platyrrhini	Wild
Howler monkey	Alouatta pigra	El Chal, Dolores, Peten, Guatemala	2008	E, N, O, P	22		Wild
Tamarin	Leontocebus weddelli	Madre de Dios, Peru	2010 to 2011	R, V	39		Wild
	Saguinus imperator		2011		36		Wild
Titi monkey	Plecturocebus cupreus	California National Primate Research Center, CA, USA	2007	O, R, V	131		Captive

a Sample types are as follows: R, rectal; F, fecal; O, oral; N, nasal; E, ear; V, vaginal; P, penile; A, axillary.

### Primate microbiome variation. (i) All host species, all body sites.

**FIG 1:**
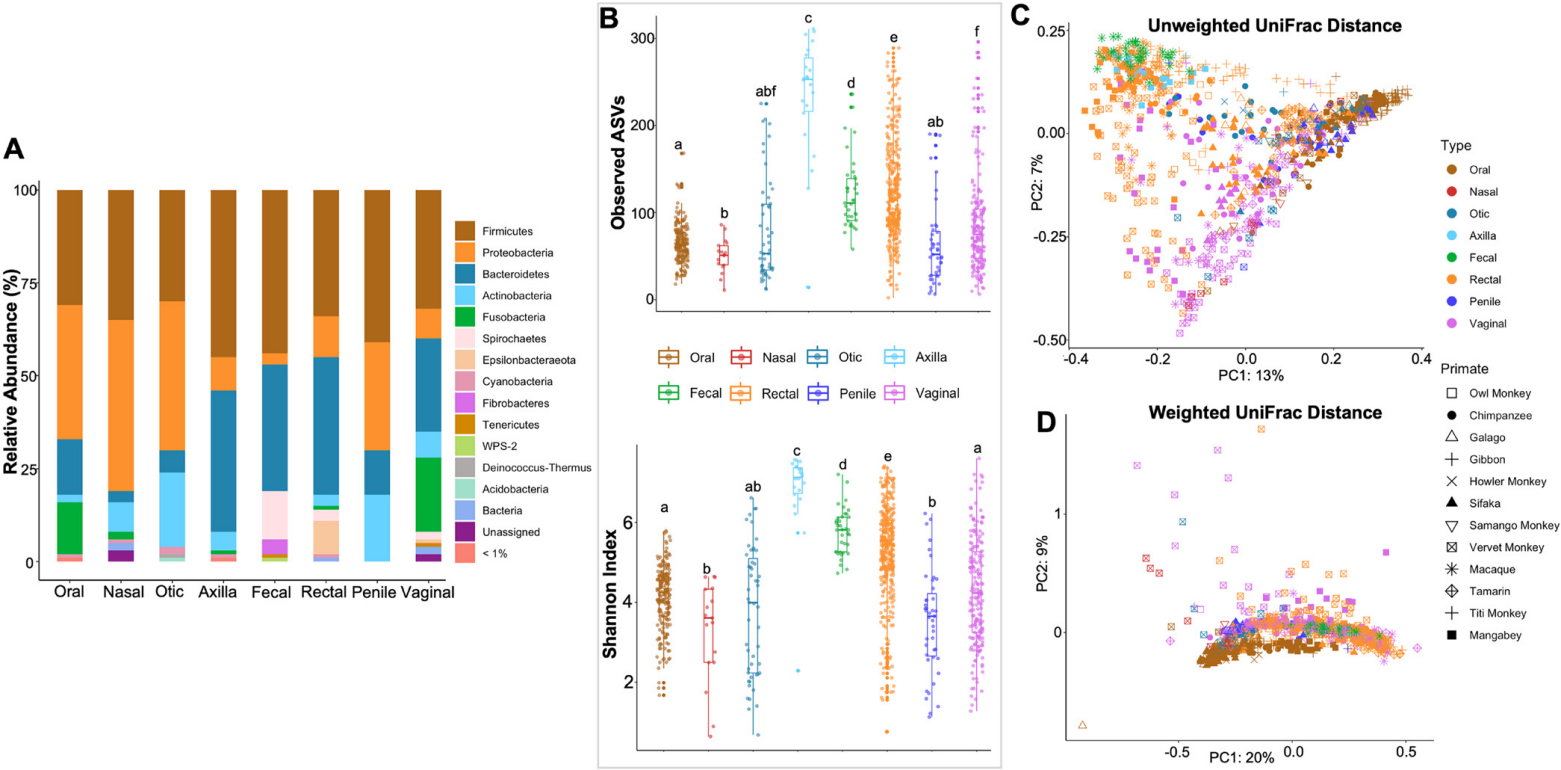
(A) Relative abundances (%) of phylum-level microbial community compositions of different body sites for all host species combined. (B) Alpha (within-sample) diversity showing the species richness and evenness of all samples. Boxplots of observed ASVs (a qualitative measure of community richness) and Shannon’s diversity index (a quantitative measure of community richness). Results marked with the same letter are not statistically significantly different at an alpha value of 0.05, while results with different letters are statistically significantly different at an alpha value of 0.05. (C and D) Beta (between-sample) diversity, showing the distribution of samples. (C) PCoA plot of unweighted UniFrac distances (a qualitative measure of community dissimilarity that incorporates phylogenetic relationships between the microbial species). (D) PCoA plot of weighted UniFrac distances (a quantitative measure of community dissimilarity that incorporates phylogenetic relationships between the features).

We first analyzed microbial communities based on all 898 samples. At the phylum level of classification, samples from the various body sites were dominated, to various degrees, by relatively high levels of *Firmicutes*, *Bacteroidetes*, *Proteobacteria*, and *Actinobacteria* (Fig. 1A). There were also specific phyla that showed relatively high levels in some of the body sites (Fig. 1A). For example, the oral, nasal, otic, and penile samples were made up of relatively large amounts of *Proteobacteria*, whereas the oral, axillary, fecal, rectal, and vaginal samples also showed relatively high levels of *Bacteroidetes*. Other examples included large amounts of *Fusobacteria* in the oral and vaginal samples, *Actinobacteria* in otic and penile samples, *Spirochaetes* in fecal samples, and *Epsilonbacteraeota* in the rectal samples. These results showed clear differences in the relative abundances of microbial taxa from the different body sites.

Differences in the microbiota compositions among samples from the different body sites were summarized with various alpha diversity measures, including observed amplicon sequence variants (ASVs), Shannon index, Faith’s phylogenetic diversity (PD), and Pielou’s evenness (Fig. 1B; Fig. S2A and B). The body sites with the lowest alpha diversities included nasal, oral, penile, and otic, whereas the axillary, vaginal, and rectal samples had the highest alpha diversities (Fig. 1B; Fig. S2A and B). Kruskal-Wallis tests showed statistically significant differences among the alpha diversities of the various body sites (*P *< 0.0001 for all measures of alpha diversity) (Table S3). Pairwise comparisons also showed these significant differences, except for the following body site pairs: fecal versus rectal, nasal versus penile, oral versus vaginal, and otic versus nasal, oral, penile, or vaginal (Table S3). We have also reported the effect sizes, which ranged from small to large (Table S3). In almost all cases, pairwise comparisons with statistically significant *P* values were also associated with moderate to large effect sizes (Table S3).

The results for beta diversity among all samples showed clustering particularly by body site (unweighted UniFrac, *F*_7,847_ = 30.84; *r*^2^ = 0.16; *P *= 0.001) (Fig. 1C and D; Fig. S2C; Table S3), as well as by host species (unweighted UniFrac, *F*_11,847_ = 15.68; *r*^2^ = 0.13; *P *= 0.001) (Fig. 1C and D; Fig. S2C; Table S3) and body site-host interaction on the microbiota composition (unweighted UniFrac, *F*_32,847_ = 4.53; *r*^2^ = 0.11; *P *= 0.001). Most notably, the oral microbiomes of all primate species clustered closely together and distinctly separate from all other body site microbiomes among all hosts.

### (ii) Host species with oral and other samples.

**FIG 2:**
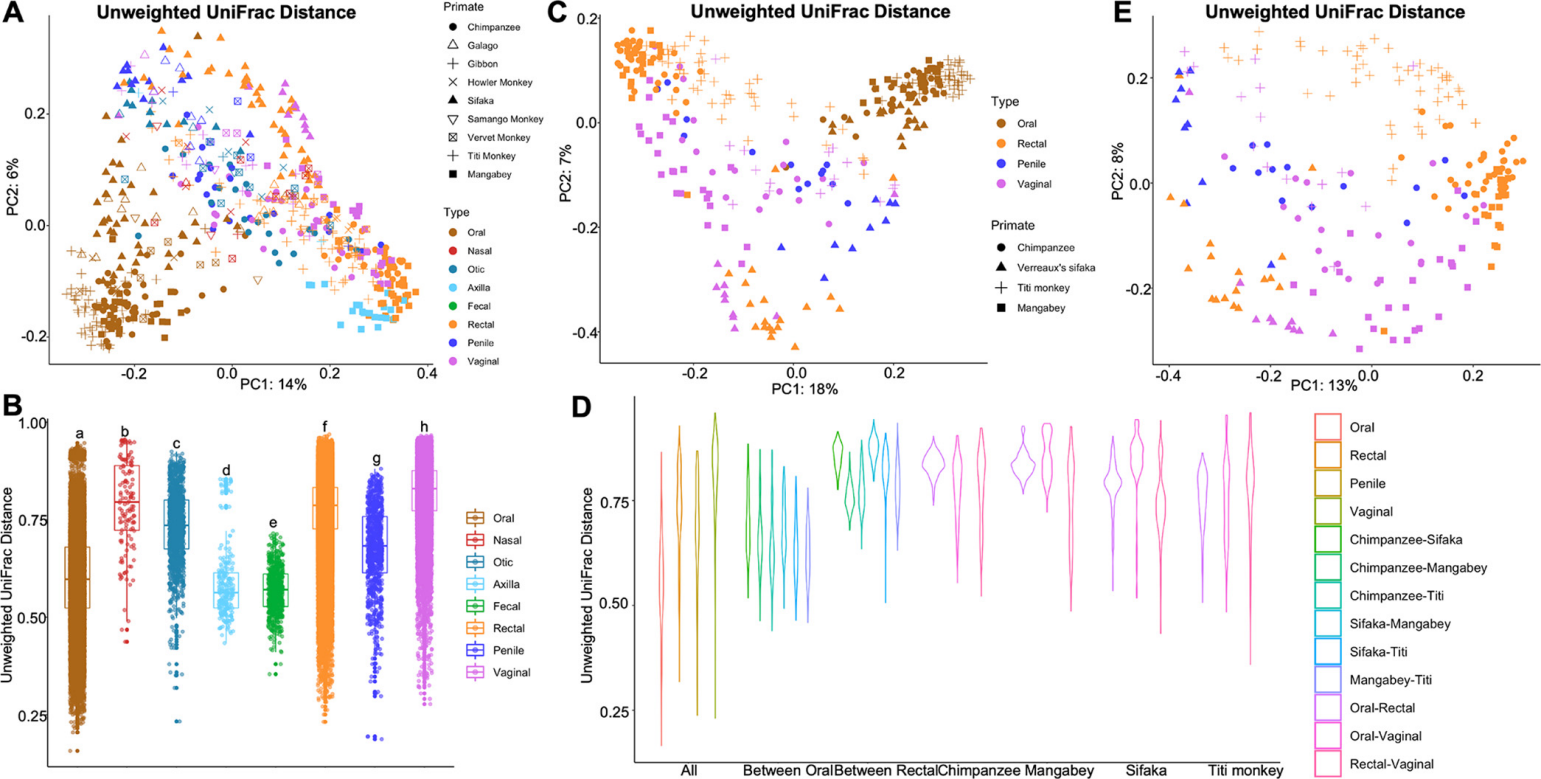
(A and B) Beta (between-sample) diversity, showing the distribution of samples, for samples from all species contributing oral samples and samples from at least one other body site. (A) PCoA plot of unweighted UniFrac distance (a qualitative measure of community dissimilarity that incorporates phylogenetic relationships between the microbial species). (B) Boxplot of quantified interindividual distances within all body sites. Results marked with the same letter are not significantly different at an alpha value of 0.05, while results with different letters are significantly different at an alpha value of 0.05. (C and D) Oral, rectal, and penile/vaginal samples from chimpanzee, Verreaux’s sifaka, mangabey, and titi monkey. (C) Beta (between-sample) diversity, showing the distribution of samples. PCoA plot of unweighted UniFrac distances (a qualitative measure of community dissimilarity that incorporates phylogenetic relationships between the microbial species). (D) Boxplot of the quantified beta diversity distances within and between groups. (E) Rectal, penile, and vaginal samples from chimpanzee, Verreaux’s sifaka, mangabey, and titi monkey. Beta (between-sample) diversity, showing the distribution of samples. PCoA plot of unweighted UniFrac distances (a qualitative measure of community dissimilarity that incorporates phylogenetic relationships between the microbial species).

To further examine the observed pronounced clustered bifurcation pattern of the oral and other body site microbiomes more robustly, we reduced potential confounders due to differential host and body site sampling. The subsequent analysis compared all host species for which we had oral samples and samples from at least one other body site. This more stringent analysis revealed even clearer separation of oral samples from all other body site samples, as seen in both unweighted and weighted UniFrac distance metrics results (Fig. 2A; Fig. S2C).

The permutational multivariate analysis of variance (PERMANOVA) results supported the statistically significant principal effects of body site (unweighted UniFrac, *F*_6,547_ = 26.10; *r*^2^ = 0.17; *P *= 0.001) (Table S4) and, to a lesser extent, host species (unweighted UniFrac, *F*_8,547_ = 11.88; *r*^2^ = 0.10; *P *= 0.001) (Table S4) and their interaction (unweighted UniFrac, *F*_29,547_ = 4.18; *r*^2^ = 0.13; *P *= 0.001) (Table S4) on the microbiota composition. The quantified interindividual variation between samples from each body site seen in the boxplot showed in general a wide range of variations within most of the body sites, including oral, rectal, and vaginal samples. The lowest interindividual variations were observed in the fecal, axillary, and nasal samples (Fig. 2B). The pairwise body site comparisons using PERMANOVA showed statistically significant differences between the interindividual distances of the different body sites (*P *= 0.001 for all measures of beta diversity) (Fig. 2B; Table S3).

### (iii) Matched samples within four host species.

Whereas the previous analyses optimized sample diversity across host species and body sites to elucidate patterns of variation, the subsequent analyses tested the rigor of these results by optimizing sampling consistency. These analyses controlled for host and body site sampling variation by comparing matched samples from three body sites (oral, rectal, and genital [penile/vaginal]) of the same individual for chimpanzees (*n* = 47), mangabeys (*n* = 23), titi monkeys (*n* = 47), and sifakas (*n* = 19), each representing one of the four Primates clades (hominoid, cercopithecoid, platyrrhines, and strepsirrhines, respectively). Bolstering the results described above, both weighted and unweighted UniFrac distances upheld the distinct oral microbiome clustering pattern (Fig. 2C; Fig. S3A to C). The results of the PERMANOVA tests supported statistically significant effects of body site (unweighted UniFrac, *F*_3,317_ = 34.09; *r*^2^ = 0.188) (Table S5) and, to a lesser degree, host species (unweighted UniFrac, *F*_3,317_ = 19.60; *r*^2^ = 0.11) (Table S5) and their interaction on the microbiota composition (unweighted UniFrac, *F*_7,317_ = 9.61; *r*^2^ = 0.12; *P *= 0.001) (Table S5).

Consistent with the results from all samples described above, we observed in general a wide range of variations in the oral, rectal, penile, and vaginal samples from the matched sample set analysis (Fig. 2D, All). While there were wide variations in all body sites, the lowest beta diversity was seen in the oral samples (unweighted UniFrac, *P *= 0.001) (Fig. 2D, All; Table S5). This is further evidenced by the statistically significant lower interindividual distances in the oral samples between host species compared to the interindividual distances in the rectal samples between host species (unweighted UniFrac, *P *= 0.001) (Fig. 2D, Oral and Rectal; Table S5).

To further establish how closely related the oral samples were, we compared the interindividual distances in the oral samples between host species to pairwise comparisons across body sites within host species (Fig. 2D). There were lower overall distances within the oral samples than in the pairwise body site comparisons within each host species (unweighted UniFrac, *P *= 0.001) (Fig. 2D, Oral in Chimpanzee, Sifaka, Mangabey, and Titi monkey; Table S5). Notably, this further supported the finding that oral microbiomes across multiple host species were more similar than samples from different body sites within the same host (Fig. 2D).

### (iv) Oral samples removed.

Removing oral samples from the analyses resulted in a pattern of clustering due to both body site and host species (Fig. 2E; Fig. S3B). The PERMANOVA results showed statistically significant effects of both body site (unweighted UniFrac, *F_2_*_,_*_202_* = 11.53; *r*^2^ = 0.08; *P *= 0.001) (Table S6) and host species (unweighted UniFrac, *F_3_*_,_*_202_* = 19.44; *r*^2^ = 0.19; *P *= 0.001) (Table S6) and their interaction on microbiota composition (unweighted UniFrac, *F_4_*_,_*_202_* = 5.80; *r*^2^ = 0.08; *P *= 0.001) (Table S6). When the oral samples were removed, however, host species, not body site, appeared to be the main factor driving the clustering observed, as seen from the higher *r*-squared values (Table S6). These results confirmed the conclusion that the oral samples predominantly drove the divergent clustering patterns seen (e.g., Fig. 1C and D and Fig. 2A and C; Fig. S2C and 3A), whereas without the oral samples, host species differences accounted for most of the variation in the microbiota compositions (Fig. 2E; Fig. S3B).

### (v) Niche differentiation.

**FIG 3:**
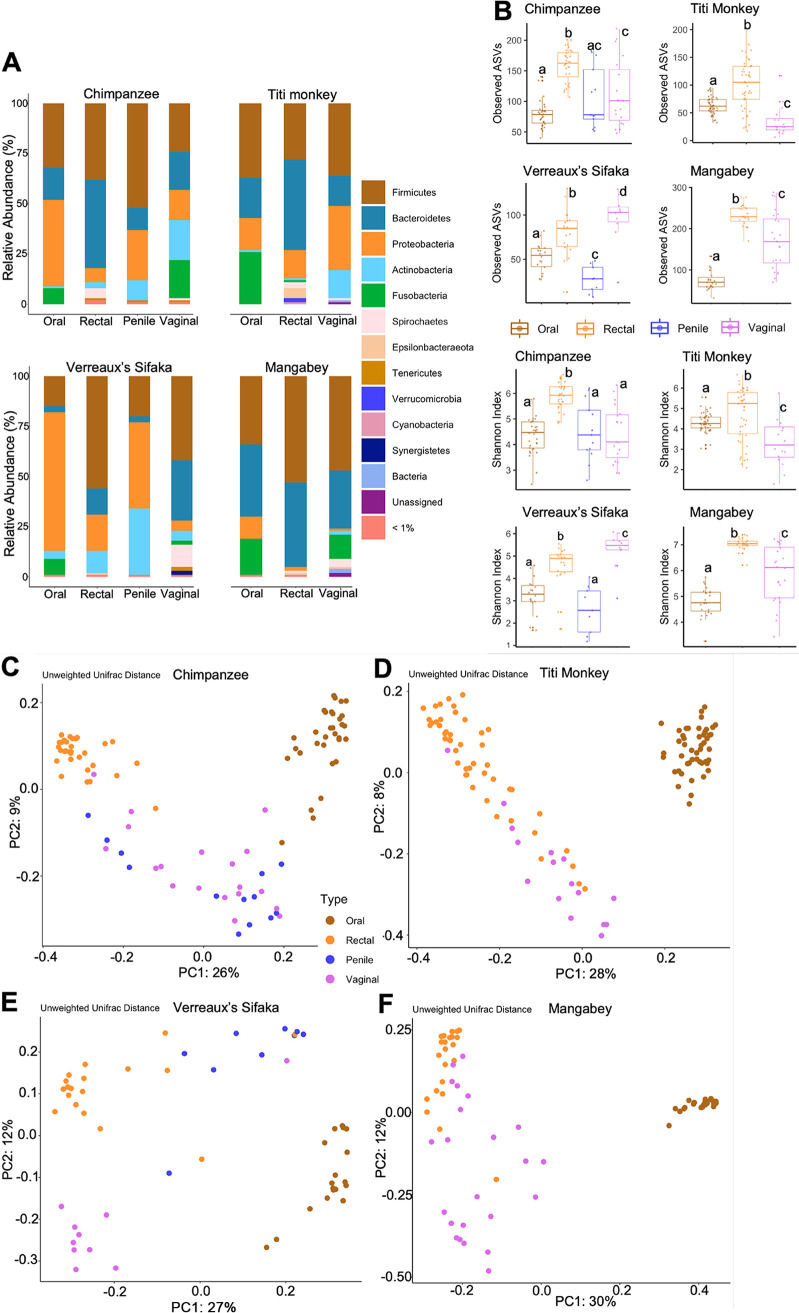
Oral, rectal, penile, and vaginal samples from chimpanzees, Verreaux’s sifakas, mangabeys, and titi monkeys. (A) Phylum-level microbial community compositions of samples. (B) Alpha (within-sample) diversity, showing the species richness and evenness of samples. Boxplots of observed ASVs (a qualitative measure of community richness) and Shannon’s diversity index (a quantitative measure of community richness). Results marked with the same letter are not significantly different at an alpha value of 0.05, while results with different letters are significantly different at an alpha value of 0.05. (C to F) Beta (between-sample) diversity, showing the distribution of samples. PCoA plots of unweighted UniFrac distances (a qualitative measure of community dissimilarity that incorporates phylogenetic relationships between the microbial species) for chimpanzee (C), titi monkey (D), Verreaux’s sifaka (E), and mangabey (F).

In assessing niche specialization, primate samples were found to be dominated at the phylum level of classification by *Firmicutes*, *Bacteroidetes*, *Proteobacteria*, *Fusobacteria*, and *Actinobacteria* in amounts that varied depending on host species and body site (Fig. 3A). For instance, chimpanzee and sifaka oral samples were dominated by *Proteobacteria*, while mangabey oral samples were dominated by *Firmicutes* and *Bacteroidetes*. Titi monkey vaginal samples appeared to have little to no *Fusobacteria*, which is found in the other species’ vaginal samples in appreciable amounts.

These differences were further seen in the microbial community richness and evenness of the various body sites within each host species (Fig. 3B; Fig. S4A). We observed the lowest and highest alpha diversities in oral and rectal samples of both chimpanzees and mangabeys, respectively (Fig. 3B; Fig. S4A). We also observed the lowest and highest alpha diversities in vaginal samples of titi monkeys and sifakas, respectively (Fig. 3B; Fig. S4A). In general, the sifakas had lower microbial species richness than the other host species (Fig. 3B; Fig. S4A). The Kruskal-Wallis test showed an overall statistically significant difference in the alpha diversities of the various body sites within each host species (*P *< 0.001 for all measures of alpha diversity except for evenness [titi monkey]) (Table S7). These results were supported by the pairwise comparisons, which also showed statistically significant differences between all pairs (*P *< 0.01 for all measures of alpha diversity) (Table S7) except the following: oral versus penile (all hosts), oral versus vaginal (chimpanzee, mangabey, and titi monkey), penile versus vaginal (chimpanzee and titi monkey), and rectal versus vaginal (titi monkey). These results were further confirmed by the large effect sizes associated with statistically significant results (Table S7).

We carried out further analyses to determine microbial community variations within and between the body sites of each of these four host species. Principal-coordinate analysis (PCoA) plots of all measures of beta diversity showed a general clustering by body site, mainly driven by oral samples in all four primate species (Fig. 3C to F, Chimpanzee, Titi Monkey, Verreaux’s sifaka, and Mangabey, respectively; Fig. S4B to E), echoing the findings of the comprehensive data set described above. The overall and pairwise comparisons using PERMANOVA showed statistically significant differences between the beta diversities of the different body sites within each host species (*P *= 0.001 for all measures of beta diversity) (Table S7).

## DISCUSSION

This comparative survey of microbial communities among eight body sites across 17 nonhuman primate species represents, to our knowledge, the first and largest comparative study with such diversity in both host species and body sites. Our results reveal that across all primates, the oral microbiome is distinct and unique in comparison to microbial communities at all other body sites and potentially conserved in NHPs. We also observe that when oral samples are omitted, microbial community variation is driven more by host species differences than body site differences.

### Evidence suggests that the oral microbiome is conserved among nonhuman primates.

The results of the comparative analyses of all 898 primate samples, followed by more specific comparative tests, support the idea that, particularly for the oral microbiome, microbial niche specialization extends across host-specific boundaries. This result is remarkable especially considering the major dietary differences among primates, from obligate frugivores to folivores, gumnivores, faunivores, graminivores, and omnivores. Despite these considerable differences and additional host-specific, genetic, phylogenetic, and environmental factors, oral microbial communities across all NHPs cluster closely together and singularly diverge distinctly from all other body sites. These results are consistent with results from previous work comparing the oral, anal, and vaginal microbiomes of wild macaques and humans, which reveals a relatively conserved oral microbiome between the two host species (27). Dental calculus sampled from historical remains of *Alouatta*, *Gorilla*, *Pan*, archeological Neanderthals, and modern humans (21) also shows support for a core oral microbiome. Within the cluster of the oral samples, there is subclustering by host species, consistent with previous results for host-specific impacts on oral (28), vaginal (14, 20), and fecal (1–4) microbiomes. These results are further supported by our PERMANOVA analysis, which shows a significant impact of host species-body site interaction on the microbiome.

The generalized oral sampling protocol used across all samples in this study permits comparisons across host species. As Huttenhower et al. (24) identified nine sites in the mouth, each with a distinct microbial community, it is fair to consider that the range of variation observed in the quantified pairwise distances in the oral microbiome may be influenced by the general sampling protocol utilized in the mouth. Both alpha (Faith’s PD) and beta (UniFrac distances) metrics account for phylogenetic relationships between the microbial species and imply that the microbial taxa observed in the oral microbiome are evolutionarily conserved among NHPs. Strong selective pressure may explain the conserved oral microbiome among diverse NHP species. Fermentation in the large intestine requires specific microbes corresponding to factors like host diet, digestive physiology, and genetics, while microbial composition and metabolic activity in the oral cavity are influenced mainly by the oral environment, including saliva and host enzymes (29, 30). Saliva acts as a buffer system to protect the oral cavity by rendering the oral environment less conducive for potentially pathogenic microbes (31–33). Saliva also contains several proteins with antibacterial properties, such as amylases, immunoglobulins (Ig), lactoferrin, mucins, histatins, peroxidases, lysozymes, and cystatins, which further impact the microbial composition of the oral cavity (31–33). Oral bacterial biofilms also provide substantial resistance to change agents. These high selective pressures and protective measures present in the oral environment appear to be conserved across host species, reducing the effect of host-specific factors on the oral microbial composition. Follow-up comparative analyses are under way to assess the functional capacities of oral microbes across diverse host species and to assess whether the oral microbiota is also divergent from all other body site microbial communities and evolutionarily conserved in other mammalian hosts.

### Host species influences the nonhuman primate microbiome.

Analyses of all samples, followed by systematic analyses of subsets of samples, support the idea that primate microbiomes differentiate not only by body site but also by host species, particularly so when the oral microbiome is excluded. Our PERMANOVA tests show that both factors significantly shape microbial community composition. The removal of the oral samples, however, results in clustering by host species, indicating that the clustering by body site observed is predominantly due to the oral microbiomes. This is further supported by the PERMANOVA results, which show that more of the microbial variation is explained by host species differences when oral samples are omitted. These results further build on our results of clustering by host species within the oral microbiome discussed above. Host-specific factors have been reported to be significant determinants of primate microbiome composition in fecal (1–4), oral (28), and vaginal (14, 20) microbiomes. These results, based on samples from multiple body sites of multiple host species, support the importance of host-specific factors on an even larger scale.

Microbiome studies of specific body sites have shown that the associated microbial communities are characterized by one or a few signature microbial taxa, mainly due to niche specialization, e.g., fecal (1–5, 15, 19, 34), oral (28, 35–38), and vaginal (14, 20) microbiomes. And yet, notably, the extent of this finding varies considerably among body sites and within versus between host species, emphasizing the importance of considering the relative levels of variation (within versus among hosts, species, and body sites) for understanding the factors affecting microbial variation. The evidence for niche specialization is even more apparent in this study of multiple body sites and host species comparisons that show more similarities among samples from the same body site than from different body sites within the same host species, consistent with other studies (22–24, 27).

Another factor considered to influence patterns of microbial variation is the impact of captivity on the microbiome composition of the different body sites and host species. While this was not directly examined here, one can surmise from prior studies that captivity impacts body site microbial communities differently. For example, while the gut microbiome has been shown to be significantly influenced by captivity (8, 9), Yildirim et al. (14) did not find an effect of captivity on the vaginal microbiomes of diverse primate species in their comparative study. It is clear from these results that the significance of captivity for the microbiome, like other factors, depends on the type of sample under consideration. While captivity may not impact the vaginal or oral microbiome significantly, it appears to be a significant factor impacting variation in the gut microbiome and supports both the differential patterns of resilience and stability and the selective pressures of endogenous and exogenous factors on microbial communities and their hosts.

### Conclusions.

This large, multiple body site and host species comparative microbiome analysis supports that the NHP oral microbiome is wholly distinct from all other body site microbial communities and conserved across diverse primate species despite their considerable genetic, dietary, habitat, and phylogenetic differences. The relatively lower interindividual distances within oral samples in comparison to other body site samples support the conserved nature of the oral microbiota across the Primates order and suggest that this is also the case in our own species’ evolutionary history.

Across all body sites and host species, the differences among microbial communities are thus predominantly driven by the distinctiveness of the oral microbiome from all other body sites.

Excluding the oral samples reveals the importance of host species-specific effects on microbial community composition across body sites. Within host species, niche specialization influences the microbial composition, as seen by the microbial similarities across primates in samples from the same body site.

Interspecies and body site comparative studies such as this are imperative to better understand factors affecting patterns of variation in host microbiomes, provide evolutionary insights into host-microbe origins, and help to contextualize and put into perspective the relative and absolute differences in microbiome variation due to body site and host species. Further intra- and inter-host species microbiome comparisons and metagenomic analyses will help to elucidate comparative microbial functions and the effects of internal (host genetics and phylogeny, digestive physiology etc.) and external (diet, environment, geography, and social) factors impacting microbial variation, with broader human evolutionary, biological, and health implications.

## MATERIALS AND METHODS

### Ethics statement.

The methods used for noninvasive collection of microbial samples from wild and captive primates were reviewed and approved by the Institutional Animal Care and Use Committee (IACUC) at the University of Illinois at Urbana-Champaign and by corresponding committees of the institutions where collaborators who contributed samples work. This research adhered to the American Society of Primatologists “Principles for the Ethical Treatment of Non-Human Primates.”

### Samples.

Our data set included 925 samples, including fecal samples and rectal, oral, nasal, otic/ear, vaginal, penile, and axillary swabs collected from 17 nonhuman primate species (approximately 50 samples/species, mainly obtained from the wild), representing nine families from the four primate clades (hominoids, cercopithecoids, platyrrhines, and strepsirrhines). Primate sample data are summarized in Table 1. Not all body sites were sampled from every individual and host species. However, the breadth and depth of the sampling allowed a range of analyses to (i) describe comparative variations among and within both body sites and host species and (ii) permit more refined analyses with larger samples sizes while controlling for host species and/or body site.

### Sample collection.

Sampling protocols and collection supplies were provided to all collaborators. The protocols requested that fecal samples (2 to 5 g per animal) be collected opportunistically from the center of the fecal bolus immediately after deposition. Sterile swabs (Copan Diagnostics, Corona, CA, USA) were used to collect microbial samples from the oral, nasal, otic, and vaginal cavities, as well as from the penis and axilla. For oral samples, the oral cavity was swabbed by gently rotating the swab in the oral cavity, representing a generalized sampling instead of a site-specific sampling. Vaginal samples were collected as described in Yildirim et al. (14). Each sample was immediately placed in a sterile 5-mL Falcon tube containing 2 to 3 mL RNAlater and mixed well to maintain sample integrity. All samples were frozen at the end of the day and shipped to the Carl R. Woese Institute for Genomic Biology at the University of Illinois at Urbana-Champaign, where they were immediately transferred into −80°C freezers until DNA extraction.

### Genomic DNA extraction.

DNA extraction and isolation from swabs was done using the QIAamp DNA minikit (catalog number 51304; Qiagen, Inc.), following the manufacturer’s protocol. Briefly, swabs stored at −80°C were thawed and transferred into 1.5-mL microcentrifuge tubes containing 500 μL phosphate-buffered saline (PBS). Samples were then centrifuged for 10 min at 13,000 × *g* (7,500 rpm), and the supernatant discarded. The swab and cell pellet were then resuspended in a lysis solution (400 μL of PBS, 180 μL buffer ATL, 20 μL proteinase K, and 200 μL buffer AL, supplied in the QIAamp DNA minikit), which was then mixed by vortexing for 15 s and incubated at 56°C for an hour. Following incubation, 200 μL ethanol (96 to 100%) was added to the digested samples and vortexed briefly. The digest was run through the QIAamp mini-spin columns (supplied in the QIAamp DNA minikit), where the genomic DNA was captured on the silica membrane. The sample was then washed by adding 500 μL buffer AW1 and centrifuging at 13,000 × *g* (7,500 rpm) for 1 min, followed by 500 μL buffer AW2 and centrifuging at 13,000 × *g* (7,500 rpm) for 1 min, and eluted in a 50-μL volume of elution buffer.

DNA from the fecal samples was extracted using the QIAamp PowerFecal DNA kit (catalog number 51304; Qiagen, Inc.), following the manufacturer’s protocol, after washing samples in 500 μL 1× PBS to remove the RNAlater in which they were stored. Briefly, approximately 0.25 g of fecal sample was added to the PowerBead tubes with 750 μL of PowerBead solution and homogenized using the MP Bio FastPrep-24 (MP Biomedicals, Solon, OH, USA) for 60 s at 6.0 m/s to obtain a more effective and rapid lysis of the cells instead of vortexing. We completed the subsequent steps of the DNA extraction as outlined in the manufacturer’s protocol. Genomic DNA was then quantified on a Qubit fluorometer (Life Technologies, Grand Island, NY) using the high-sensitivity double-stranded DNA (dsDNA) kit.

### 16S rRNA hypervariable region sequencing.

We amplified the hypervariable V3-V5 region of the 16S rRNA gene using PCR primer set 357F (5′-CCTACGGGAGGCAGCAG-3′) and 926R (5′-CCGTCAATTCMTTTRAGT-3′) from the Human Microbiome Project (39) as follows: extracted genomic DNA samples were sent to the Roy J. Carver Biotechnology Center at the University of Illinois at Urbana-Champaign for amplicon library synthesis on the Fluidigm Access Array system. Using the high-sensitivity DNA kit, the concentration of each DNA sample was measured on a Qubit fluorometer (Life Technologies) and diluted to 2 ng/μL. A mastermix for amplification was prepared using the Roche high-fidelity fast-start kit and 20× Access Array loading reagent according to Fluidigm protocols.

The first round of PCR was completed with a reaction mixture consisting of 4 μL of mastermix, 2 μL of each primer, and 2 ng of DNA sample in 1 μL nuclease-free water using the following conditions: 50°C for 2 min, 70°C for 20 min, and 95°C for 10 min; 10 cycles of 15 s at 95°C, 30 s at 55°C, 1 min at 72°C; 2 cycles of 15 s at 95°C, 30 s at 80°C, 30 s at 60°C, 1 min at 72°C; 8 cycles of 15 s at 95°C, 30 s at 55°C, 1 min at 72°C; 2 cycles of 15 s at 95°C, 30 s at 80°C, 30 s at 60°C, 1 min at 72°C; 8 cycles of 15 s at 95°C, 30 s at 55°C, 1 min at 72°C; and 5 cycles of 15 s at 95°C, 30 s at 80°C, 30 s at 60°C, 1 min at 72°C.

The harvested primary PCR product was then transferred to a new 96-well plate and diluted 1:100 in water, and 1 μL of diluted product was added to 15 μL of reagent mix and 4 μL of Illumina linker barcodes for a second round of amplification. This 20-μL reaction mixture was denatured for 10 min at 95°C and amplified with 14 cycles of 15 s at 95°C, 30 s at 60°C, and 1 min at 72°C, with a 3-min extension at 72°C. All libraries were quantified on a Qubit fluorometer and run on a Fragment Analyzer (Advanced Analytics, Ames, IA), and amplicon regions and expected sizes were confirmed. Samples were then pooled in equal molar amounts, size selected on a 2% agarose E-Gel (Life Technologies), and extracted from the isolated gel slice with the Qiagen gel extraction kit (Qiagen). Cleaned size-selected products were run on an Agilent Bioanalyzer to confirm appropriate profile and determination of average size.

The final 16S amplicon pools were quantitated using the Qubit fluorometer (Life Technologies, Grand Island, NY), further quantitated by quantitative PCR (qPCR) on a Bio-Rad CFX Connect real-time system (Bio-Rad Laboratories, Inc. CA), and then pooled evenly. The pool was then denatured, spiked with 20% nonindexed PhiX control library provided by Illumina, and loaded onto the MiSeq version 2 flow cell at a concentration of 8 pM for cluster formation and sequencing on the MiSeq. The libraries were sequenced from both ends of the molecules to a total read length of 250 nucleotides from each end.

### Data analyses.

Using the sample-specific barcodes assigned during sequencing, the sequenced data were demultiplexed in QIIME2-2018.11 (https://qiime2.org) (40). Quality control, denoising, chimera removal, and amplicon sequence variant (ASV) generation were completed using the Divisive Amplicon Denoising Algorithm (DADA2) (41) for each sequence run. Taxonomy was then assigned at 99% similarity based on the SILVA taxonomy and reference database (SILVA_132_QIIME_release) (42). A rooted phylogenetic tree was built using the “align-to-tree-mafft-fasttree” pipeline from QIIME2.

The processed sequenced data were then used to compare the microbial community composition, richness, and diversity among samples. Microbiome comparisons were based on sets of primate host species for which we had samples from the same body sites (Table 1). We assessed the microbial community taxonomic composition of the samples by assigning taxonomy as outlined above and generating relative-abundance bar plots based on totals per group of samples. To select the optimal sampling depth for rarefaction purposes, we tested several depths using the “qiime diversity alpha-rarefaction” script and selected a depth of 1,500 for all subsequent analyses. We also completed alpha (within-sample variation) and beta (between-sample variation) diversity analyses with the “qiime diversity core-metrics-phylogenetic” script, which rarefied the different feature tables to the specified sampling depth of 1,500 and computed various alpha (observed ASV, Pielou’s evenness, Shannon index, and Chao1 index) and beta (weighted and unweighted UniFrac) diversity metrics (43). Using the “qiime diversity alpha-group-significance” script, we then tested the associations between body site and host species and alpha diversity results. In addition to *P* values, we also computed the effect size for the Kruskal-Wallis test as the eta squared based on the H statistic (eta^2^[H]), with the following interpretations: 0.01 to <0.06, small effect; 0.06 to <0.14, moderate effect; and ≥0.14, large effect. We then used the “qiime diversity beta-group-significance” script to determine the interindividual distances within and between groups. The pairwise option also completes pairwise tests to determine which specific pairs of groups are similar. We then generated boxplots and principal-coordinate analysis (PCoA) plots from these results using the QIIME2R package in R (R software, version 3.6.0).

We also tested for statistically significant differences in sample clustering patterns and microbial community composition due to host species and body site using permutational analysis of variance (PERMANOVA; adonis function in the vegan package, R software, version 3.0.6). This tests for the amount of statistical variation explained by the factors and their interactions (*r*^2^ and *F* values, which show the strength of the effects) and whether the differences due to these factors and/or their interactions are statistically significant (*P* value) (44).

To explore how the different sampling regimens (e.g., differences in samples sizes and body sites sampled on animals of different species) may have affected the results due to smaller sample sizes (e.g., nasal and otic) or uneven distribution of host species per body site, analyses were conducted at various comparative levels (e.g., total sample set, all samples from one body site, and matched individual host-body sites across a phylogenetically controlled subset of species from each of the major primate clades [hominoids, cercopithecoids, platyrrhines, and strepsirrhines]).

### Data availability.

Raw sequencing files can be found on the SRA database (BioProject accession number PRJNA795815).
