# Supplementary material for: Large Comparative Analyses of Primate Body Site Microbiomes Indicate that the Oral Microbiome Is Unique among All Body Sites and Conserved among Nonhuman Primates
Source: Microbiol Spectr. 2022 May 19;10(3):e01643-21. doi: 10.1128/spectrum.01643-21 (PMC9241786; doi:10.1128/spectrum.01643-21)
Supplement: SUPPLEMENTAL FILE 2 — Fig. S1 to S4. Download spectrum.01643-21-s001.pdf, PDF file, 0.9 MB [file spectrum.01643-21-s002.pdf]

Supplementary Figures

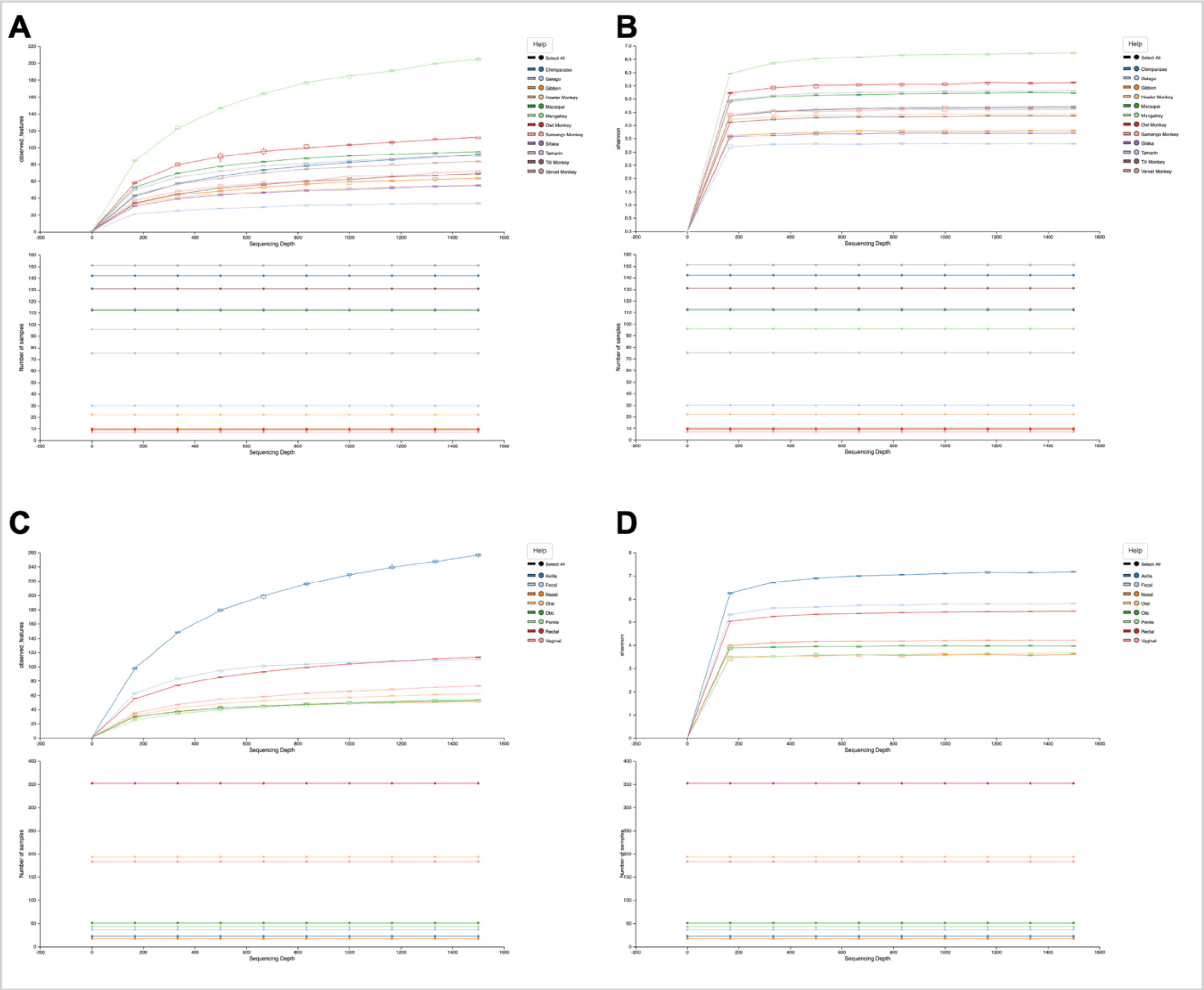

Supplementary Figure 1

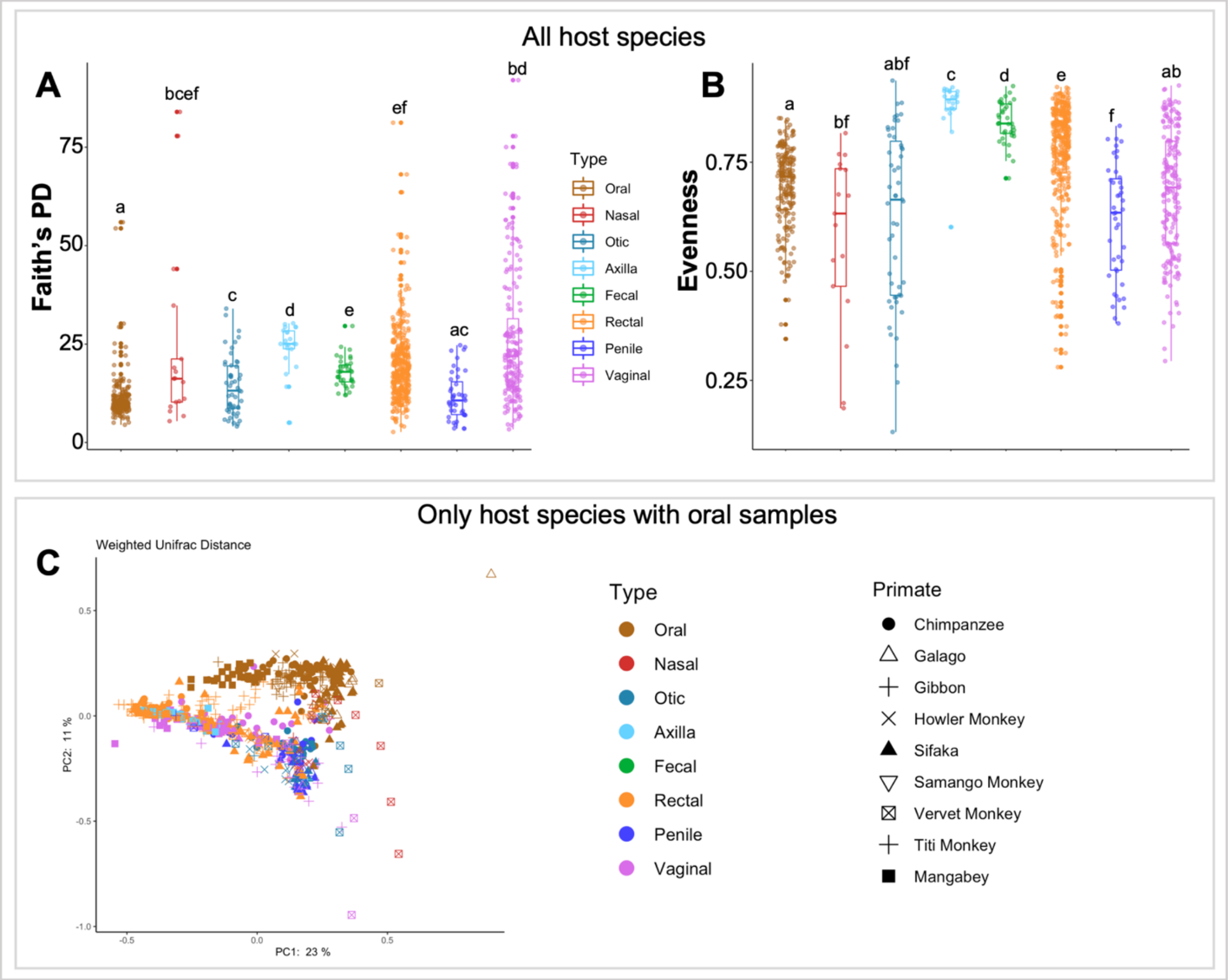

Supplementary Figure 2

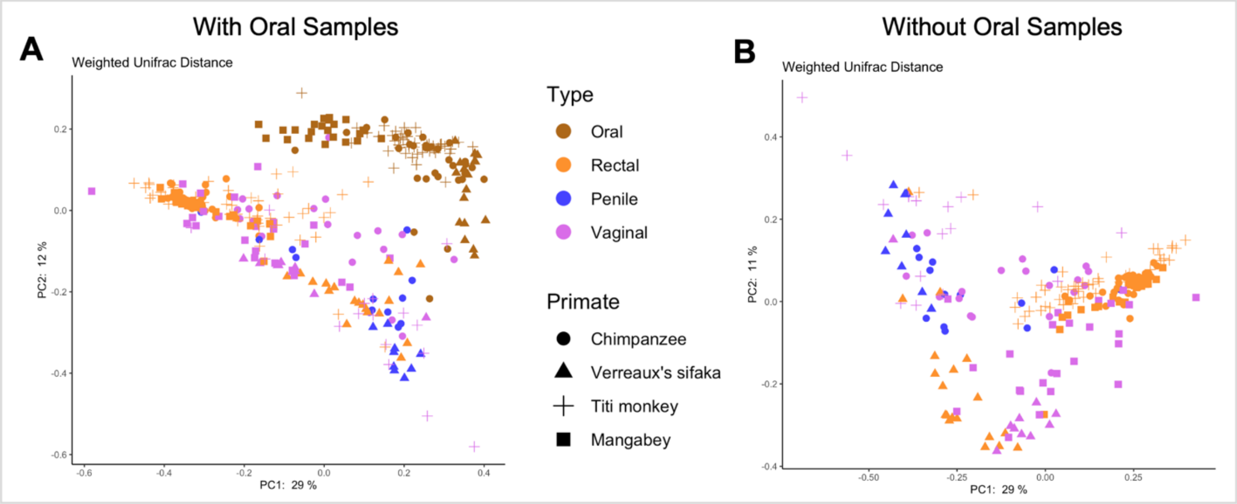

Supplementary Figure 3

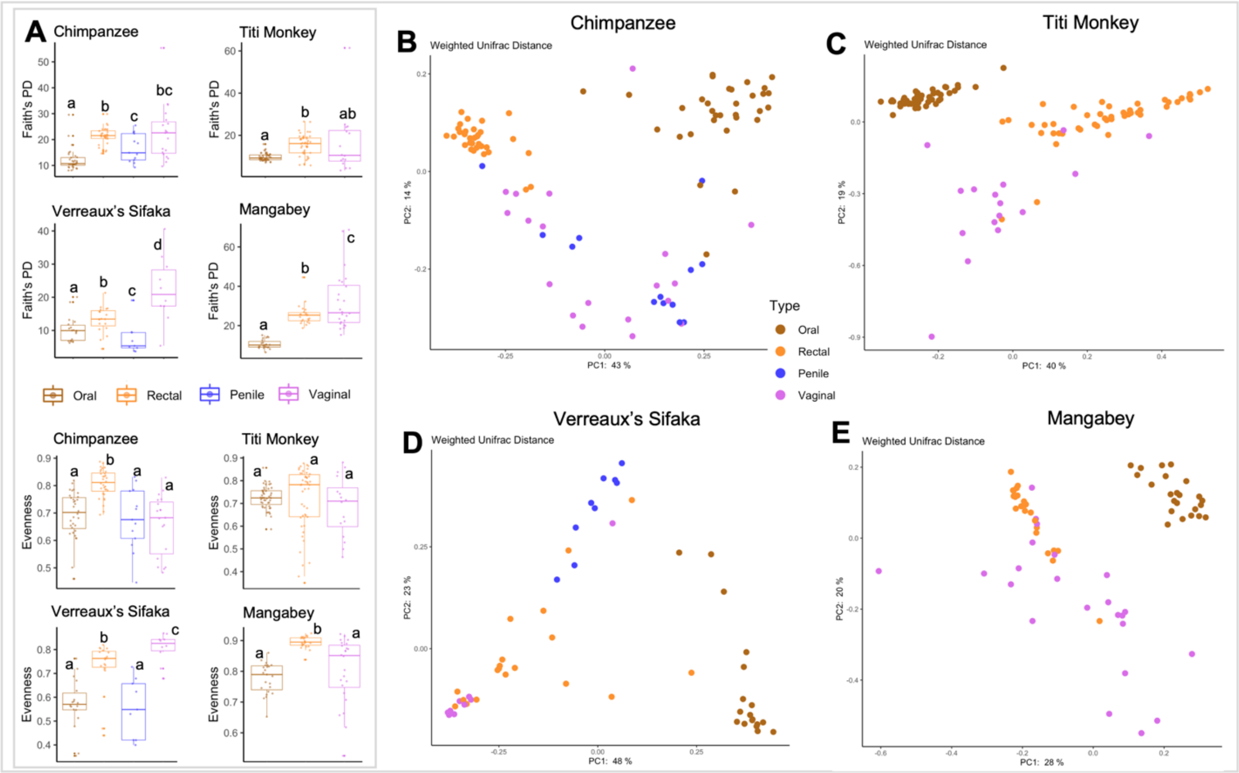

Supplementary Figure 4

Legends

Supplementary Figure 1: All samples from all host species. Alpha rarefaction. Alpha diversity as a function of sampling depth A: Observed ASVs (a qualitative measure of community richness) and B: Shannon's diversity Index (a quantitative measure of community richness) grouped by host species. Alpha diversity as a function of sampling depth C: Observed ASVs (a qualitative measure of community richness) and D: Shannon's diversity Index (a quantitative measure of community richness) grouped by body site

Supplementary Figure 2: A – B: Alpha (within-sample) diversity showing the species evenness of all samples: Boxplots from A: Faith's Phylogenetic Diversity (a qualitative measure of community richness that incorporates phylogenetic relationships between the microbial species) and B: Evenness (a measure of community evenness). C: Beta (between-sample) diversity showing the distribution of samples: PCoA plots from weighted UniFrac distances (a quantitative measure of community dissimilarity that incorporates phylogenetic relationships between the features).

Supplementary Figure 3. A: Samples: oral, rectal, penile, and vaginal samples from chimpanzee, Verreaux's sifaka, mangabey and titi monkey. Beta (between-sample) diversity showing the distribution of samples: PCoA plots from weighted UniFrac distances (a quantitative measure of community dissimilarity that incorporates phylogenetic relationships between the features).

B: Samples: rectal, penile, and vaginal samples from chimpanzee, Verreaux's sifaka, mangabey and titi monkey. Beta (between-sample) diversity showing the distribution of samples: PCoA plots from weighted UniFrac distances (a quantitative measure of community dissimilarity that incorporates phylogenetic relationships between the features).

Supplementary Figure 4: Samples: oral, rectal, penile, and vaginal samples from B: chimpanzee, C: titi monkey, D: Verreaux's sifaka and E: mangabey. A: Alpha (within-sample) diversity showing the species richness and evenness of samples: Boxplots from Faith's Phylogenetic Diversity (a qualitative measure of community richness that incorporates phylogenetic relationships between the microbial species) and Evenness (a measure of community evenness). B – E: Beta (between-sample) diversity showing the distribution of samples: PCoA plots from weighted UniFrac distances (a quantitative measure of community dissimilarity that incorporates phylogenetic relationships between the features.
